# 2018 Update on Protein-Protein Interaction Data in WormBase

**DOI:** 10.17912/micropub.biology.000074

**Published:** 2018-11-26

**Authors:** Jaehyoung Cho, Christian A Grove, Kimberly Van Auken, Juancarlos Chan, Sibyl Gao, Paul W Sternberg

**Affiliations:** 1 Division of Biology and Biological Engineering 156-29, California Institute of Technology, Pasadena, CA 91125, USA; 2 Informatics and Bio-computing Platform, Ontario Institute for Cancer Research, Toronto, ON M5G0A3, Canada

**Figure 1:**
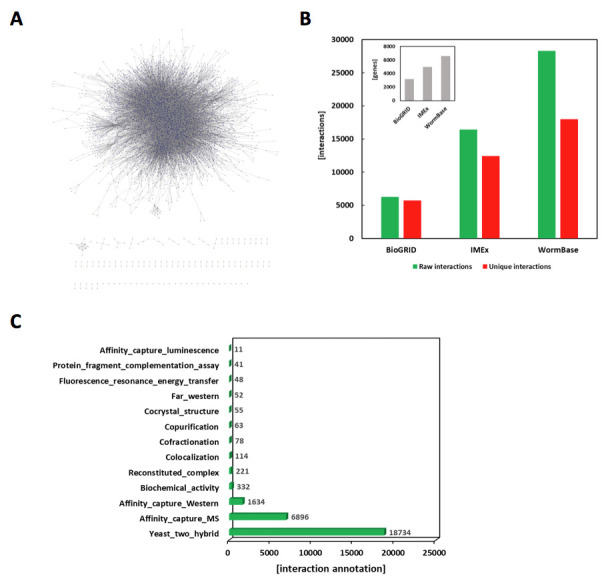
**(A)** Network diagram showing a map of *C. elegans* protein-protein interactions. This cluster includes 17,990 unique protein-protein interactions from 6,079 protein coding genes in the *C. elegans* genome. This map was generated by Cytoscape 3.6.1. **(B)** Comparisons of the curation status between the major databases for *C. elegans* protein-protein interaction data. As of September 2018, IMEx has 16,443 total and 12,433 unique protein-protein interactions with 4,967 unique genes annotated from 253 papers. BioGRID has 6,274 total and 5,734 unique protein-protein interactions with 3,212 unique genes annotated from 174 papers. In contrast, WormBase has 28,279 total and 17,990 unique protein-protein interactions with 6,079 unique genes annotated from 1,251 papers. The inserted bar-graph shows the number of unique genes curated for the protein-protein interactions in each database. **(C)** Statistics of the experimental evidence types curated for the protein interaction data set in WormBase. The experimental evidence type for each protein interaction is annotated as described in the BioGRID curation guide for ‘physical experimental systems’ (https://wiki.thebiogrid.org/doku.php/curation_guide:biochemical_experimental_systems). However, in WormBase, ‘Colocalization’ is annotated as an evidence type only when the interaction is supported by other detection methods. Within the total 18,734 yeast two-hybrid interactions, 17,312 interactions come from large-scale, high-throughput studies and 1,422 come from small-scale studies.

## Description

Protein interaction is an important data type to understand the biological function of proteins involved in the interaction, and helps researchers to deduce the biological nature of unknown proteins from the well-characterized functions of their interaction partners. High-throughput studies, coupled with the aggregation of individual experiments, provides a global ‘snapshot’ of the protein interactions occurring at all levels of biological processes or circumstances. This snapshot of the interaction network, the interactome, is important to understand the overall events up to the level of comparison between species or pathway simulation, or to find new factors yet undefined in the processes, or to add details to the biological processes and pathways.

As of September 2018, WormBase (www.wormbase.org) (Lee *et al.* 2018) contains 28,279 physical protein-protein interactions for the roundworm *Caenorhabditis elegans*. Among these, 1500 protein-protein interactions have been curated by BioGRID as a collaboration with WormBase. Within the data set, 17,990 protein-protein interactions are unique, and 6,079 unique genes are involved in these interactions. In order to visualize the overall interaction map, a network diagram for all the unique interactions was generated by using the ‘Cytoscape’ program, version 3.6.1 (Shannon *et al.* 2003) (Figure 1A). These numbers represent a 108% increase in the number of interaction annotations since last year, 2017. These interaction data were curated from 1,251 peer-reviewed papers, which were selected from the literature by ‘Textpresso Central’ using automatic SVM (Support Vector Machine)-based text mining approaches (Fang *et al.* 2012; Müller *et al.* 2018) and manual verification. Compared to other databases providing *C. elegans* protein-protein interaction, WormBase now presents the largest data set, which has 1.72-fold more interaction annotations than IMEx (Orchard *et al.* 2012) and 4.51-fold more than BioGRID (Chatr-Aryamontri *et al.* 2017) (Figure 1B). Most significantly, WormBase now houses the complete protein interaction data from almost all of the *C. elegans* literature published from 1993 to 2018. The data sets presented at IMEx and BioGRID are annotated from 253 and 174 papers, respectively. All the physical interaction data in WormBase are supported by experimental evidence from original research papers. The statistics of the detection methods used as experimental evidence are shown in Figure 1C. The majority of the interaction data came from high throughput analysis such as large-scale yeast two-hybrid assays or mass-spectrometry, however, a significant portion of the data (13.1%) are supported by more direct detection methods using small-scale, low throughput methods such as co-immunoprecipitation or co-crystallography (Figure 1C).

In WormBase, protein-protein interaction data can be found as a subclass of physical interaction data in the ‘Interactions widget’ on the gene report page. The Interactions widget provides all types of interaction data related to the gene of interest, such as physical, genetic, regulatory, and predicted interactions. All the interaction data are represented together in a graph created with ‘Cytoscape.js’ and a table. In the table, the gene names of interaction partners (bait-target) in the interaction are displayed along with the publication. The interaction details including the detection method are also captured in the summary and the remark field in the Interactions page. Users can query the data by using the search bar on the WormBase front page or download all the available data files from the WormBase FTP site (ftp://ftp.wormbase.org/pub/wormbase/releases/current-production-release/species/c_elegans/PRJNA13758/annotation/c_elegans.PRJNA13758.WSXXX.interactions.txt.gz, where WSXXX is the database version release, like “WS267”).

All the interaction data in WormBase will be available soon at the new information resource for multiple model organisms, the Alliance of Genome Resources (https://www.alliancegenome.org/). This site will integrate all the interaction data from human and from model organisms *C. elegans*, budding yeast (*Saccharomyces cerevisiae*), fruit fly (*Drosophila melanogaster*), zebrafish (*Danio rerio*), mouse (*Mus musculus*) and rat (*Rattus norvegicus*). Integrated views of interaction data from diverse model organisms will be extremely helpful to build interaction databases for species-to-species comparison, and to establish a disease model quickly based on the database. For the most efficient analysis of the interaction data in WormBase, we are now working on developing a new ‘Venn diagram tool’ and integrating the ‘Gene Set Enrichment Analysis tool’ (https://wormbase.org/tools/enrichment/tea/tea.cgi) into the Interactions widget. We will continue to curate other types of macro-molecular interactions including protein-DNA, protein-RNA and RNA-RNA interactions, as well as newly reported protein-protein interaction data to serve our research community.

## Reagents

All the interactions data are available at the WormBase FTP site(ftp://ftp.wormbase.org/pub/wormbase/releases/current-production-release/species/c_elegans/PRJNA13758/annotation/c_elegans.PRJNA13758.WSXXX.interactions.txt.gz, where WSXXX is the database version release, like “WS267”).
